# Exploring the roles of informal healthcare providers in urban settings to improve access and quality of care for the urban poor: experience from Bangladesh, Ghana, Nepal, and Nigeria

**DOI:** 10.1093/heapol/czag087

**Published:** 2026-07-08

**Authors:** Dominic Dormenyo Gadeka, Bryony Dawkins, Noemia Teixeira de Siqueira Filha, Asiful Haidar Chowdhury, Abhigyna Bhattarai, Chinyere Mbachu, Md Badruddin Saify, Henry Okudzeto, Mattheus Rodrigues Da Silva, Sushil Chandra Baral, Zahidul Quayyum, Genevieve Cecilia Aryeetey, Rumana Huque, Obinna Onwujekwe, Bassey Ebenso, Helen Elsey, Irene A Agyepong

**Affiliations:** Department of Health Policy, Planning and Management, University of Ghana School of Public Health, P.O. Box LG13, Legon, Accra, Ghana; Academic Unit of Health Economics, Leeds Institute of Health Sciences, University of Leeds, Leeds LS2 9NL, United Kingdom; Department of Health Sciences, Hull and York Medical School, University of York, York YO10 5DD, United Kingdom; ARK Foundation, Dhaka, Bangladesh; HERD International, Lalitpur, Nepal; Health Policy Research Group, University of Nigeria, Enugu PMB 01129, Nigeria; ARK Foundation, Dhaka, Bangladesh; Department of Health Policy, Planning and Management, University of Ghana School of Public Health, P.O. Box LG13, Legon, Accra, Ghana; Graduate Program in Economics-PIMES, Federal University of Pernambuco, Recife, Brazil; HERD International, Lalitpur, Nepal; BRAC James P Grant School of Public Health, BRAC University, Dhaka, Bangladesh; Department of Health Policy, Planning and Management, University of Ghana School of Public Health, P.O. Box LG13, Legon, Accra, Ghana; ARK Foundation, Dhaka, Bangladesh; Health Policy Research Group, University of Nigeria, Enugu PMB 01129, Nigeria; Nuffield Centre for International Health and Development, Leeds Institute of Health Sciences, University of Leeds, Leeds LS2 9NL, United Kingdom; Hull and York Medical School, University of York, York YO10 5DD, United Kingdom; Faculty of Public Health, Ghana College of Physicians and Surgeons, P.O. Box MB 429, Accra, Ghana

**Keywords:** informal healthcare providers, urban poor, urban health, quality of care, low- and middle-income countries

## Abstract

Though informal healthcare providers (IHPs), often defined as unregistered and not formally trained, are heavily utilized by low-income urban residents and present an opportunity to address the urban primary care gap, these providers are rarely prioritized or integrated into health systems. This paper explores IHPs’ role in urban settings across four rapidly urbanizing low- and middle-income countries: Bangladesh, Nepal, Nigeria, and Ghana, to enhance understanding of their potential within health systems and promote their integration to improve access and quality of care for the urban poor. We studied published and unpublished articles and policy documents to compare informal provider types, roles, services, and target populations in each country. We then present case studies illustrating how these providers support healthcare for urban poor. A narrative synthesis was applied to describe the included documents/literature and results, while quantitative data were analysed using STATA version 16. OpenStreetMap basemaps were employed to illustrate informal providers’ locations within urban areas. Our findings show that across the four countries, there is substantial evidence that informal providers contribute significantly to health service delivery by bridging the care gap, particularly for socially and economically disadvantaged urban populations. They serve as the first point of contact, offering a wide range of health services, including preventive, curative, and restorative services. However, they are loosely defined and lack robust standards, protocols, professional training, and accreditation. Informal providers play a critical, though varied, role in the delivery of healthcare across the countries studied, facilitating access to care where it might otherwise be unavailable or unaffordable. Initiatives to strengthen the informal sector, e.g. linking or integrating with the formal sector, have the potential to improve healthcare access and quality for the urban poor.

Key messages

- More than half of the population in low- and middle-income countries reside in cities where healthcare access is generally assumed to be better than in rural areas. However, a significant portion lives in impoverished slum areas that face substantial barriers to access quality and affordable primary healthcare.
- There is substantial evidence across the four countries studied that informal healthcare providers significantly contribute to health service delivery by bridging this care gap, offering access where care might otherwise be unavailable or unaffordable.
- However, there are concerns about the quality of care in the informal sector, as these providers lack robust standards, protocols, professional training, and accreditation, despite delivering extensive health services.
- Initiatives such as linking or integrating informal providers with the formal sector could enhance healthcare access and quality for the urban poor.

## Introduction

Health systems continue to grapple with global challenges posed by urbanization, which often occurs rapidly and without control in low- and middle-income countries (LMICs). Although more than half of the population in LMICs resides in cities, where healthcare access is generally assumed to be better, a significant portion lives in impoverished areas facing substantial barriers to quality and accessible primary healthcare (PHC; Mundia and Murayama 2010, Ezeh et al. 2017, Lilford et al. 2025). The infrastructure of these cities, particularly public primary care facilities, is largely ill-equipped to meet the evolving health needs of a large and expanding urban population, placing it under considerable strain (Adams et al. 2018, Elsey et al. 2019, Lilford et al. 2025). Additionally, the urban poor, defined as socially and economically disadvantaged populations, often encounter financial difficulties in paying for health services, limited coverage of health services, and unfavourable operating hours (de Siqueira Filha et al. 2022). In addition, the opening hours of PHC facilities usually conflict with the extended workdays characteristic of low-income daily wage earners (Mundia and Murayama 2010, Ezeh et al. 2017). Consequently, in many LMICs, the urban poor are often compelled to depend on informal healthcare providers (IHPs) for their health needs (Ozor et al. 2025). However, reliance on IHPs is not driven solely by formal healthcare system constraints. In many of these urban LMIC contexts, IHPs are preferred because they have long-standing relationships with community members, have built trust over time, share social and cultural norms, and are perceived as responsive to patient needs (Sudhinaraset et al. 2013, Adams et al. 2015, Thapa et al. 2021, Kumah 2022).

While formal healthcare providers (FHPs) are recognized by a country’s regulatory and legal framework, there is no single definition of an IHP. Common attributes include providers that offer primary care services without formal recognition and regulation, or with little or no formal professionally recognized training (Sudhinaraset et al. 2013, Clarke et al. 2019, Thapa et al. 2021). The characteristics of IHPs may vary depending on the context; however, they include traditional birth attendants (TBAs), patent medicine vendors (PMVs), traditional healers, faith healers, drug sellers, bonesetters, herbalists, and native doctors (Sieverding and Beyeler 2016, Kumah 2022, Odii et al. 2024, Onuh et al. 2024). Nonetheless, studies have consistently shown the reliance of the urban poor in many countries in sub-Saharan Africa and South Asia on IHPs as the first point of contact for people in need of healthcare, including preventive, curative, and restorative services (Onwujekwe et al. 2011, Adams et al. 2015, Mackintosh et al. 2016, Kumah 2022, Mbachu et al. 2024). The estimated utilization rate of IHPs in LMICs ranges from 9% to 90% (Sudhinaraset et al. 2013, Thapa et al. 2021).

While it is crucial to acknowledge the important role of IHPs within their communities in improving coverage and accessibility of health services by filling gaps in healthcare provision, reports on the quality of the services they provide are often mixed (Sieverding and Beyeler 2016, Ezeh et al. 2017, Kruk et al. 2018, Odii et al. 2024). Quality of care is ‘the degree to which health services for individuals and populations increase the likelihood of desired health outcomes and are consistent with current professional knowledge’ (WHO 2018). High-quality care involves providing effective, safe, people-centred healthcare that is timely, equitable, integrated, and efficient. The services by IHPs are often characterized by poor supervision, medical errors, non-adherence to national clinical guidelines (e.g. inappropriate and illegal prescribing and dispensing of drugs), lack of referrals as well as gaps in knowledge and provider practices, and increased avoidable mortality incidents (Sudhinaraset et al. 2013, Sieverding and Beyeler 2016, Ezeh et al. 2017, Arize et al. 2025). These pose substantial risks to individual patients and the broader health system. For instance, a significant share of antibiotic dispensing in Bangladesh, India, Zambia, and Congo is attributed to IHPs amidst the global challenge of antimicrobial resistance due to inappropriate and poor prescribing practices (Nahar et al. 2020, Ingelbeen et al. 2022, Thapa et al. 2022, Wildbret et al. 2023). Additionally, in Nigeria, the use of IHPs has been linked to delays in cancer diagnosis (Fayehun et al. 2025), limited appropriate referrals, with only 7% of private providers in informal settlements making proper referrals (Obi et al. 2025), as well as issues like polypharmacy, drug–drug interactions and toxicity (Miller and Goodman 2016), and overuse of hospitals (Elsey et al. 2019). Furthermore, an earlier study, found that only 9% of IHPs possess adequate knowledge of hypertension and diabetes (Mbachu et al. 2024). However, this situation is particularly dire, as having a non-communicable disease (NCD) can result in two to seven times higher odds of incurring catastrophic out-of-pocket costs (Jan et al. 2018).

Given the plurality of IHPs and the large number of urban poor populations exposed to low-quality services, understanding their role and potential may help to identify initiatives to effectively strengthen them and to form linkages between them and FHPs to improve access to and quality care. This could also help to accelerate progress towards universal health coverage (UHC), which seeks to ensure that all people have access to the full range of quality health services they need, when and where they need them, without financial hardship (World Health Organization 2023). The importance of strengthening these linkages within health systems has long been advocated in global policies and strategies (World Health Organization 2020). However, IHPs are still not sufficiently prioritized, engaged, or integrated into the health system (Odii et al. 2024, Tandan et al. 2024, Okechi et al. 2025). Studies in Nigeria highlight how integration of the informal sector within formal healthcare is acceptable to communities, formal health system actors, and informal providers themselves (Onuh et al. 2024, Onwujekwe et al. 2025b). The World Health Organization recognizes that UHC cannot be achieved through public-sector services alone without involving the private sector, including IHPs (World Health Organization 2020). Evidence abounds that linkages between formal and IHPs greatly improve access to and quality of health services, including better diagnosis, treatment, waiting times, length of stay, referral rates, mortality rates, and patient satisfaction (Shaikh et al. 2014, Muzyamba et al. 2017, Basabih et al. 2022). For instance, in Zambia, it was found that when TBAs were trained, they provided counselling, referral, and logistical support, including treatment adherence support to formal care providers (Muzyamba et al. 2017). Also, there is evidence that when IHPs are engaged with appropriate support and training, they identify tuberculosis (TB) patients and refer and administer Direct Observed Treatment to TB patients (Thapa et al. 2021).

In this paper, we explored the roles of IHPs within urban contexts across four rapidly urbanizing LMICs: Bangladesh, Nepal, Nigeria, and Ghana. Across these countries, there is a significant role for IHPs in the provision of healthcare, but there are differences in the way informal providers are understood, and their services are utilized. We conducted a document and literature review to summarize and compare the types and roles of IHPs, the services they offer, and the populations to whom they provide services in each country. We then present illustrative case studies from each country to highlight the presence and use of IHPs and to underscore their contribution to the delivery of healthcare for the urban poor within the pluralistic urban health system. We use the term ‘case study’ to mean the presentation of data from a selected study or studies to illustrate IHPs’ presence and use in each context. By examining the role of IHPs, we aimed to provide a better understanding of this important sector to inform their potential within the health systems and promote their integration into the mainstream health system to improve access and quality of care for the urban poor.

### Conceptual framework of the role of informal healthcare providers

The study was guided by an integrated framework combining three complementary lenses: the pluralistic health systems lens (Health Market Systems framework; Bloom and Standing 2001, Bloom et al. 2008), PHC (Astana Declaration; World Health Organization 2018), and Penchansky and Thomas’ five dimensions of access: availability, accessibility, affordability, acceptability, and accommodation (Penchansky and Thomas 1981). The Health Market Systems framework views healthcare as characterized by the coexistence of public-sector providers, private formal providers, and informal providers, positioning IHPs within the existing health economy and advocating for their governance to strengthen the health system. The PHC framework highlights accessibility, continuity of care, equity, and multisectoral action, illustrating how IHPs contribute to or fall short of PHC attributes and emphasizing the need to strengthen PHC as a foundation for UHC. Penchansky and Thomas’ five dimensions of access highlight patient-centred drivers of care and provide a lens for equity and quality of care and in this context explain why the urban poor rely on IHPs despite the presence of the formal health system.

Based on these, our framework conceptualizes the role of IHPs within the pluralistic urban health systems of Bangladesh, Ghana, Nepal, and Nigeria. Rapid urbanization, urban poverty, social determinants of health, and inadequate PHC capacity create barriers to access for the urban poor. In this context, IHPs emerge as accessible, affordable first points of care, influencing healthcare utilization through their availability, affordability, and acceptability. The framework underscores the dual effects of engaging with IHPs: improved access and continuity of care, alongside potential quality risks due to weak regulation, limited training, and inadequate supervision. The framework further highlights formal-informal linkages, such as referrals, training, and regulatory engagement as critical mechanisms to enhance quality while preserving access, contributing to equitable PHC and progress towards UHC.

## Materials and methods

### Study design

This paper builds on multiple sources of evidence from Bangladesh, Ghana, Nepal, and Nigeria. These countries are at different points in their urban transition, have a range of IHPs and provide different regional settings. First, we drew on the combination of published and unpublished articles and policy documents to understand the roles of the IHPs in the urban settings. This was followed by illustrative case studies from multiple sources, including household and facility survey data, and geographic information system mapping of health facilities through urban representative cross-sectional studies from the respective countries. The study forms part of Community-led Responsive and Effective Urban Health Systems (CHORUS) multi-country research consortium programme (https://chorusurbanhealth.org/) aimed to improve understanding of and identify ways of strengthening urban PHC in LMICs.

### Study setting, data sources, and data collection

#### Documents and literature review

We conducted rapid document and literature reviews to synthesize existing evidence on IHPs in urban settings. We did not register the study, and we did not perform a methodological quality assessment, as is the case with document and literature reviews (Grant et al. 2009). The Preferred Reporting Items for Systematic Reviews and Meta-Analysis Extension (PRISMA) criteria (Supplementary File S1) were used to guide the conduct and reporting of the review (Page et al. 2021).

##### Eligibility criteria

We were guided by the general definition of IHPs as individuals or entities that provide health-related services but are not fully recognized, licensed, or regulated within a country’s formal health system and typically lack standardized, formally accredited medical or pharmaceutical training (Bloom et al. 2011, Sudhinaraset et al. 2013, Kumah 2022). Using the definition and focusing on LMICs, we defined the following inclusion and exclusion criteria for the literature and document reviews. We included published and unpublished studies, policy documents, and reports that described the definition, evolution, governance/regulation, types, roles, services, utilization, or beneficiaries of IHPs in the urban settings of the four countries, while studies and policy documents without substantive relevance to informal providers based on these key characteristics were excluded. We excluded studies and documents on IHPs that were from countries other than our four study countries, except multi-country studies that include any of the countries. All studies and documents in languages other than English were excluded.

##### Identifying relevant studies and documents

We conducted the review between January and October 2025. We searched four electronic bibliographic databases: PubMed/MEDLINE, Web of Science, Scopus, and Google Scholar, in addition to targeted searches of grey literature and institutional sources, including the World Health Organization publication and technical reports, Ministries of Health websites for Bangladesh, Ghana, Nepal, and Nigeria; National Statistical Services and Health Regulatory Agency websites for policies and research reports. To capture the full scope of relevant studies and documents and to trace the evolution of IHPs in each country, we imposed no date restrictions on our search. The search terms combined keywords related to ‘informal healthcare providers’, ‘urban health’, ‘primary healthcare’, quality of care’, or ‘access to care’ with country-specific terms. Logical operator-based combinations of key terms were used. The final database search is presented in Supplementary File S2.

##### Study selection

We screened the titles and abstracts of the retrieved articles and documents manually against the eligibility criteria. After duplicates were removed, the full text of the selected titles and abstracts was assessed by country teams for eligibility. In cases of disagreement, the team discussed the issues until a consensus was reached. The first author further performed a manual search to identify additional studies from the reference lists of relevant publications. This included targeted searches in Google and Google Scholar. This was done by entering the full title of the text into the search engine and retrieving the full text, where available.

##### Charting the data

We developed a search strategy tool in Excel to extract the data. The extraction was conducted by each country team and validated by country team leaders for the study. To ensure consistency across heterogeneous sources, we strictly adhered to the inclusion and exclusion criteria. We resolved all disagreements through discussions at the country and/or consortium level until a consensus was reached, enabling the integration of the heterogeneous sources into a coherent narrative overview of the evidence. We discussed the results and continuously iteratively updated the data charting form.

##### Collating, summarizing, and reporting

**Figure 1:**
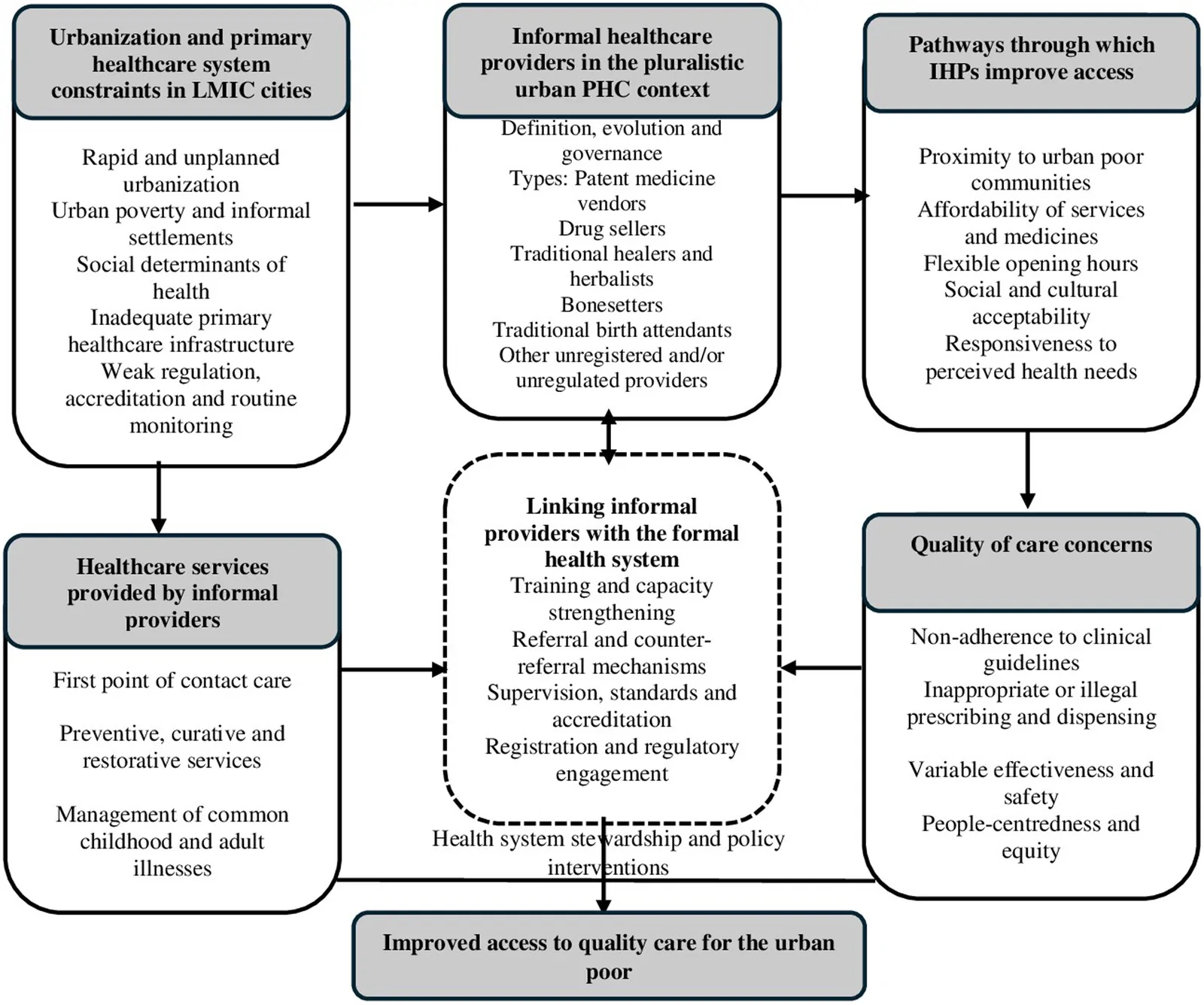
Conceptual framework of the role of IHPs in improving access to quality care for the urban poor.

We applied narrative synthesis to describe the included studies and documents and their results. Details of the final list of studies and documents reviewed are provided in Supplementary File S3. Through deductive processes based on the conceptual framework (Fig. 1), we extracted, coded, and categorized evidence to identify the role of IHPs in urban settings of Bangladesh, Ghana, Nepal, and Nigeria. As it is the focus of this paper, we only reported on the role of IHPs. Thus, we did not report data on the linkage of IHPs with the formal health system but argue for their integration into the mainstream health system to improve access and quality of care for the urban poor based on the identified roles. The charted data were mapped, and similar data were grouped to establish commonality of IHPs and their roles across different countries’ contexts. Upon completing each data extraction and analysis step, the team reviewed and discussed the findings, which are presented as tables.

#### Primary datasets (case studies)

Based on the findings from the review, we conducted country case studies to illustrate IHPs’ presence to inform their potential within the urban health systems. This included facility surveys in Nigeria and Bangladesh, household and facility surveys in Ghana, and a household survey in Nepal (all described below).

##### Bangladesh

The study mapped all drug sellers in the urban area of Mirpur in the North City Corporation of Dhaka. Mirpur is one of Dhaka's most densely populated areas and has neighbourhoods divided into 15 wards (Wards 2–16; Bangladesh Bureau of Statistics 2025). The Research Electronic Data Capture (REDCap) system, installed on Global Positioning System (GPS)-enabled tablets, was used to record the drug sellers, their names, and exact GPS coordinates. A total of 1100 drug sellers were identified and mapped, while 400 were randomly selected for data on the healthcare providers’ socio-demographic characteristics, qualifications, training, and drug dispensing knowledge, attitudes, and practices.

##### Nigeria

A facility survey was conducted in eight densely populated urban slums in Enugu and Anambra states in southeastern Nigeria. The providers were eligible if they were any type of formal or informal provider that had been delivering any type of healthcare services within the study slum sites for at least the past 6 months, other than those informal providers who offered spiritualism- or exorcism-based services. This included sole operator providers and providers with multiple members of staff. Data were collected on the type of healthcare providers and ownership across 255 facilities.

##### Ghana

The household survey involved a multistage stratified cluster sampling (*n* = 1683) while the facility survey was a census (*n* = 267) conducted in the La-Nkwantanang Madina Municipal Assembly (LaNMMA) of Ghana. Data were collected on health service utilization, including community members’ preferences for health facilities, and the factors influencing their choice of health facilities, as well as the type of services provided by health facilities. Household members who lived within the randomly sampled households were interviewed while the health facility managers responded to the facility survey.

##### Nepal

A household census was conducted in two wards of Budhanilkantha Municipality in Kathmandu, Ward 4 and Ward 7, to gather comprehensive information on demographic, socioeconomic, and health-related characteristics of the population. The data collection covered household composition, economic status, access to health facilities, health risk behaviours [such as physical activity, dietary intake (fruits, vegetables, salt smoking, and sugar sweetened beverage), and alcohol and tobacco use], and key health indicators, including maternal health, family planning, child immunization, and the prevalence of communicable and NCDs and the most preferred health facilities respondents visit when they or their family members are unwell. Special emphasis was placed on assessing risk factors for disease, such as sedentary behaviour, poor diet, tobacco and alcohol use, improper waste management, and exposure to air pollution. The census encompassed 2927 households (7097 individuals) in Ward 4 and 1733 households (4093 individuals) in Ward 7. Data were collected through structured questionnaires administered to household representatives.

### Quantitative data analysis

OpenStreetMap (OSM) basemaps were used for visualization of the drug seller facilities in Mirpur, allowing overlay of health provider locations with population density, settlement type, and health facility layers. Data from the household and health facility surveys were analysed descriptively in STATA version 16. This was descriptive and exploratory in nature and aimed to summarize patterns in the availability and role of IHPs within each country rather than to generate pooled cross-country estimates. Given the differences in data sources, study designs, and indicators across countries, analyses were conducted separately by country, and results were compared narratively across common domains. To streamline heterogeneous data points into a coherent analysis, indicators were grouped according to shared conceptual categories aligned with the study objectives, including the proportion of formal and informal health facilities available in the particular country-study context, provider preferences by the urban population, type of service being provided, and percentage of community members who utilized healthcare services by both formal and informal providers and factors that determine where the urban population seeks care. We defined the denominators based on the sampling frame and unit of analysis for each dataset. Denominators were not pooled or combined across countries and no cross-country aggregate estimates were produced. All proportions and percentages were calculated using these countries and dataset-specific denominators. Missing data were handled using complete-case analysis for descriptive reporting, and no imputation was performed (Arteaga and Ferrer-Riquelme 2009).

## Results

### Informal healthcare providers in the pluralistic urban primary healthcare context

#### Definitions, evolution, and governance

The body of literature and documents we reviewed in each country suggests that defining IHPs is challenging, as ‘formal’ providers may have ‘informal’ practices, and increasingly, countries are implementing policies to begin registering and monitoring some IHPs, which gives them some characteristics which may be used to define formal providers. However, key commonalities across countries align with the general definition as those who generally do not possess an official qualification or permit; many do not have formal medical training, and are not recognized as healthcare providers by a country’s regulatory and legal framework. We found that the extent, evolution and governance of the informal health sector varied greatly across the four countries.

In Bangladesh, the informal health sector has evolved over the last five decades. Post-1980s urbanization saw rapid migration to cities, which outpaced the formal healthcare infrastructure, including severe workforce shortages and weak urban PHC, creating demand for informal healthcare (Ahmed et al. 2011). Following this, there has been an increased peri-urban expansion where the informal providers became embedded within slum communities, often acting as first-line caregivers (Sizear et al. 2019). Despite their prevalence and important role, urban health policies have historically overlooked the informal providers. Over the past decade, however, there has been a policy shift towards acknowledging IHPs as essential components of the pluralistic health systems, through training, referral practices, and integration into disease control programmes (Bloom et al. 2011, Sudhinaraset et al. 2013). Nonetheless, most informal providers operate outside the formal regulatory frameworks of the professional councils for physicians and pharmacists, as well as national drug regulatory authorities (Ahmed et al. 2011, WHO 2015). Recent evidence highlights their role in antibiotic misuse, referral gaps, and maternal care access, prompting calls for their training and regulation (Sizear et al. 2019).

For Ghana, there has been a shift to the policy and formalization of traditional medicine with the government (Traditional Medicine Practice Council) approving the integration of traditional medical practice into the formal healthcare systems in 2012, and formal herbal medicine training offered at several universities and over 50 government hospitals have herbal medicine units (Asase 2023). To that extent, there is a Traditional and Alternative Medicine Directorate of the Ministry of Health responsible for policymaking regarding this sector (GBC Ghana 2019, Yirenkyi 2019). However, a significant portion of practitioners remain unregistered and poorly regulated (Hampshire et al. 2022, Clay 2024). Furthermore, many drug stores and chemical sellers, known as Over-the-Counter Medicine Sellers (OTCMS), are licensed to provide over-the-counter medicines, yet most practitioners lack formal training for these services (Asiedu et al. 2021). They possess limited knowledge of appropriate medical practices and treatment guidelines for various illnesses and weak regulatory compliance (Asiedu et al. 2021, Frempong et al. 2021).

In Nepal, IHPs are individuals or outlets that deliver health-related services outside the formalized, government-recognized health system. These providers often operate without official registration or authorization to practice beyond their training scope (Ban et al. 2020). They include medicine shop–based practitioners, often trained as paramedical workers (with 2–3 years of training), who operate as *de facto* clinics, diagnosing and treating patients in rural and urban areas (Ban et al. 2020). Though there has been an increase in health service provision, a significant percentage of the population still visits traditional healers (Dhami-Jhankri). The Dhami-Jhankri, and herbalists are examples of these practitioners who have been serving communities for centuries and still exist in almost every corner of the country (Subedi 2023a). These practitioners are still relevant as people consult them within the pluralistic health system, not only for physical illnesses but also for emotional or psychosocial issues (Kohrt and Harper 2008). Evidence has shown that many urban drug shops provide medical advice, dispense antibiotics without prescriptions, and offer injections and intravenous fluids (Nepal et al. 2019). Furthermore, though traditional medicine remains widely used, regulation is limited and largely voluntary (Kunwar et al. 2010).

In Nigeria, IHPs have evolved alongside traditional healing practices, with Western-style medicine introduced during European expeditions in the nineteenth century. Mission-owned hospitals were prominent before independence, and the government later organized and developed policies for general care. The emergence of organized healthcare services led to the establishment of hospitals by missionaries and the military, with a shift to centralized control by regional governments in the mid-twentieth century. However, PHC services have deteriorated in recent years, leading to medical tourism for the elite who travel out of Nigeria to seek healthcare and, for the rest of the population, a reliance on informal health providers, including patent medicine dealers, also known as PMVs, pharmacy shops, and itinerant (travelling) drug sellers (Onwujekwe et al. 2011). More recent research in urban Nigeria has further identified the important role of bonesetters, TBAs, traditional healers, and herbalists, all of whom serve the urban population, particularly those living in informal settlements (Onwujekwe et al. 2025b). The healthcare system continues to face challenges in adapting to changing disease patterns and ensuring access to quality care (Scott-Emuakpor 2013). Table 1 presents some examples of informal health providers and their definitions.

**Table 1:** Examples of IHPs and their definitions.

Informal healthcare providers	Definition
Traditional birth attendants (TBAs)	Community members who assist with childbirth and immediate care of the woman. Some TBAs have received some training to improve delivery practices and reduce maternal and infant mortality.
Bonesetters	Informal providers who manage fractures, dislocations, and congenital anomalies. They practice without formal medical training.
Patent medicine vendors	Licensed drug retailers, but the majority are not formally trained to work as pharmacists or pharmacy technicians.
Herbalists/traditional healers	Practitioners and therapeutic practices that use medicinal plants that can be associated with rituals, performed by practitioners who hold the knowledge necessary to affect the cure.

#### Types and roles of informal healthcare providers

Diverse types of IHPs were found across countries, with some commonalities. Table 2 shows the types and roles of IHPs in the four countries. While traditional healers, TBAs, bonesetters, and faith (spiritual) healers are commonly recognized as types of IHPs across the countries, allopathic providers, PMVs, homeopaths, and drug peddlers tend to be country specific, even though the nature of their services or care intentions remains similar regardless of their designation. The roles and extent of coverage by IHPs vary significantly between countries. For example, in Bangladesh, 87% of healthcare providers were informal, and in the rural area of Chakaria, this figure rose to 96% (Sudhinaraset et al. 2013). The dominants among these provides in the urban settings are the local unlicensed pharmacies and TBAs (Hossain et al. 2025). In Ghana, IHPs play a wide-ranging role, offering services from NCD screening and diagnosis, counselling, to emotional, spiritual, and psychological support, as well as maternal and child health services, including delivery, post-natal care, and treatment for fever, malaria, and diarrhoea (Tenkorang 2016, Ghana Statistical Service 2022). Similarly, in Nepal, several private health facilities operate outside the formal arrangements (Nepal et al. 2019). Non-registered pharmacies, providing both allopathic and ayurvedic medicines, traditional healers, bone setters, herbal sellers, and homeopaths, are common types of IHPs in Nepal. For example, in a comprehensive mapping of the health facilities of Pokhara Metropolitan City, 7.4% of the private health facilities were found to have been operating either without registration or without renewal (HERD International 2023). It is estimated that ∼3000 unregistered pharmacies are operating across the country (Subedi 2023b). Studies suggest that ∼60%–70% of caregivers of sick children, including mothers, seek initial care from private healthcare providers, particularly for acute respiratory infections (Bradley et al. 2018, Tiwari et al. 2022). In Nigeria, the uptake of services for drug sellers, e.g. ranges between 36% and 50% for the treatment of fever (Brieger et al. 2002, Abimbola et al. 2016).

**Table 2:** Types and roles of informal healthcare providers.

Countries	Types and roles of informal healthcare providers
Bangladesh	Include (unqualified) allopathic providers (e.g. village doctors and drugstore salespeople/drug vendors), traditional healers, non-secular faith healers, TBAs, and homeopaths (Sizear et al. 2019). A systematic review reported that 87% of healthcare providers were informal, while in the rural region of Chakaria, 96% of all providers were informal health providers. It also stated that 65% of individuals went to IHPs for care (Sudhinaraset et al. 2013).
Ghana	The IHPs in Ghana include traditional healers (herbalists) and herbal sellers, spiritual healers, bonesetters, TBA, drug peddlers, and unregistered chemical sellers. They provide a range of services, including NCD screening and diagnosis, counselling, and often serve as an alternative to biomedical/orthodox approaches to treatment and monitoring. They also offer emotional, spiritual, and psychological support. Additionally, the informal sector provides other services, including maternal and child health services, such as delivery, post-natal services, fever, malaria, and diarrhoea (Tenkorang 2016, Ghana Statistical Service 2022).
Nepal	Informal health providers include medicine shop–based practitioners, often trained as paramedical workers (with 2–3 years of training), yet operating as *de facto* clinics, diagnosing and treating patients in rural and urban areas, even though such functions fall outside their licensed scope (Ban et al. 2020). They also include traditional healers: Individuals like herbalists, spiritual or faith healers, and other community-based traditional practitioners, who rely on cultural or spiritual healing knowledge not formally integrated into biomedical systems (Subedi 2023a). Studies suggest that ∼60%–70% of caregivers of sick children, including mothers, seek initial care from private healthcare providers, particularly for acute respiratory infections (Bradley et al. 2018, Tiwari et al. 2022).
Nigeria	The informal sector includes patent medicine dealers, also known as PMVs, pharmacy shops, and itinerant drug sellers. PMVs are licensed drug retailers, but most are not formally trained to work as pharmacists (Onwujekwe et al. 2011). More recent research in urban Nigeria has further identified the important role of bonesetters, TBAs, and herbalists, all of whom serve the urban population, particularly those living in informal settlements (Onuh et al. 2024, Okechi et al. 2025, Ozor et al. 2025). The uptake of services for drug sellers ranges between 36% and 50% to treat fever in Nigeria (Brieger et al. 2002, Abimbola et al. 2016)

### Healthcare services provided by informal providers

#### Type of services provided by informal health providers

The specific services provided by IHPs in each country are extensive. To analyse and compare the types of services provided by the IHPs across countries, we classified them into four functional categories using operational definitions developed for this review: (1) long-term care, (2) ambulatory care, (3) ambulatory centres, and (4) retail services. The results are shown in Table 3. These categories were intended to capture the primary mode and intensity of care provided rather than formal facility status. We defined long-term care as ongoing or repeated care provided over an extended period, often involving chronic condition management, mental healthcare, spiritual or psychosocial support, or sustained traditional or herbal treatment. This category commonly includes traditional healers, spiritual or faith-based healers, and unorthodox mental health facilities. Ambulatory care refers to direct, episodic, face-to-face clinical, or quasi-clinical services provided without overnight admission. This included consultations, diagnosis, treatment, counselling, minor procedures, or childbirth services provided by providers such as TBAs, herbalists, bonesetters, and informal allopathic providers. Ambulatory centres were those dedicated premises where informal providers deliver services that require repeated visits or specialized focus but which do not meet the criteria for formal health facilities. Retail services refer to the sale or dispensing of medicines and health products, with or without limited clinical interaction. Providers in this category included PMVs, drug sellers, chemical sellers, and unregistered pharmacies. While some retail providers may offer advice or recommend treatments, they were classified based on their primary functions as medication supplies (based on the dominant or most frequently reported service function). In all, providers such as traditional healers, herbal sellers, and spiritual healers provide a continuum of care that ranges from ambulatory to long-term care and are dominant in Ghana and Nigeria compared with Bangladesh and Nepal. In Bangladesh and Nepal, most of the IHPs are ambulatory providers.

**Table 3:** Types of services provided by IHPs.

Countries	Types of services provided by IHPs			
	Long-term care	Ambulatory providers	Ambulatory centres	Retailers
Bangladesh	Untrained physiotherapists	TBAs, herbalists, bone setters, unqualified doctors’ chambers, Kabiraj/Hekim/Ayurbed, Traditional Peer/fakir/tantric/ojha/boi dya	Bone setters	PMVs
Ghana	Traditional healers (herbalists), herbal sellers, and spiritual healers	TBAs, traditional healers, spiritual healers, drug peddlers, chemical shops, and bonesetters	Traditional bonesetters	Traditional healers (herbalists), herbal sellers, drug peddlers, and chemical shops
Nepal	Limited; however, there is increasing practice of families hiring nurses and paramedics to provide long-term care at home.	Practice of traditional healers and TBA still exists, which is in a declining trend as government discourages such practices and outreach of formal sector providers has increased.	Practice of traditional healers and TBAs	Unregistered drug sellers
Nigeria	Unorthodox mental health facilities and use of herbal/traditional medicine	Herbalists and traditional medicine providers, including online	Bone setters and TBAs	PMVs, herbalists, and traditional medicine sellers

#### Beneficiaries of informal service provision

The category of individuals who accessed services from IHPs varies across countries. However, most of them are individuals who are socially and economically disadvantaged (Odii et al. 2024, Ozor et al. 2025). These are poor, vulnerable, and minority-indigent groups in each country. We found that females patronize the services of IHPs more compared with males, and the services are mostly tailored to childhood and adult illnesses (Table 4).

**Table 4:** Categories of people who access services from IHPs.

Countries	Category of people who access services from informal providers
Bangladesh	People, especially the poor and the disadvantaged, prefer to seek healthcare from non-qualified providers in the informal sector due to the shortage of qualified health workforce and inequitable distribution of public health providers (Ahmed et al. 2011). In the context of fragmented urban health systems, the slum dwellers in urban areas generally look for ‘quick fixes’ to avoid time off work, and seek care from the local pharmacy, considering those as ‘good enough’ for ‘common’ health issues (Heijden et al. 2019)
Ghana	Though the services of the informal providers are accessed by different segments of the population, the poor and vulnerable are the predominant service users. Compared with males, females patronize the services of the informal health providers the most (Ghana Statistical Service 2022). Often, informal providers are the first point of care for common illnesses, including communicable diseases, as well as maternal and child health services, including diarrhoea and fever, for the urban poor in densely populated urban settings, including slums. After the initial diagnosis, patients often access NCD services through formal service providers. The concept of NCDs is frequently perceived as a promise of a cure rather than a lifelong condition requiring ongoing treatment. Many patients find the idea of a cure more comprehensible than the notion of lifelong treatment, which presents challenges in maintaining motivation for adherence to treatment over the long term.
Nepal	In Nepal, informal healthcare providers provide diagnosis and treatment for patients, especially the underserved populations in rural and urban areas (Ban et al. 2020, Subedi 2023a). These practitioners are still relevant as people consult them within the pluralistic health system, not only for physical illnesses but also for emotional or psychosocial issues (Kohrt and Harper 2008). Their services have been seen to be accessible, reliable, and affordable (Subedi 2023a).
Nigeria	IHPs (PMVs, TBAs, traditional bone setters, and herbal medicine practitioners) account for a significant proportion of healthcare providers in Nigeria, delivering care, especially in under-resourced communities (Odii et al. 2024, Onuh et al. 2024). They are often the first point of care for common childhood and adult illnesses for those living in urban informal settlements in Nigeria. In communities with non-functional or inaccessible formal health systems, IHPs bridge the gap in access to healthcare services, even for households that would typically prefer to use formal (public) healthcare facilities (Arize et al. 2025).

### Section 2: illustrative country case studies of the presence of informal healthcare providers and their use

#### Bangladesh

**Figure 2:**
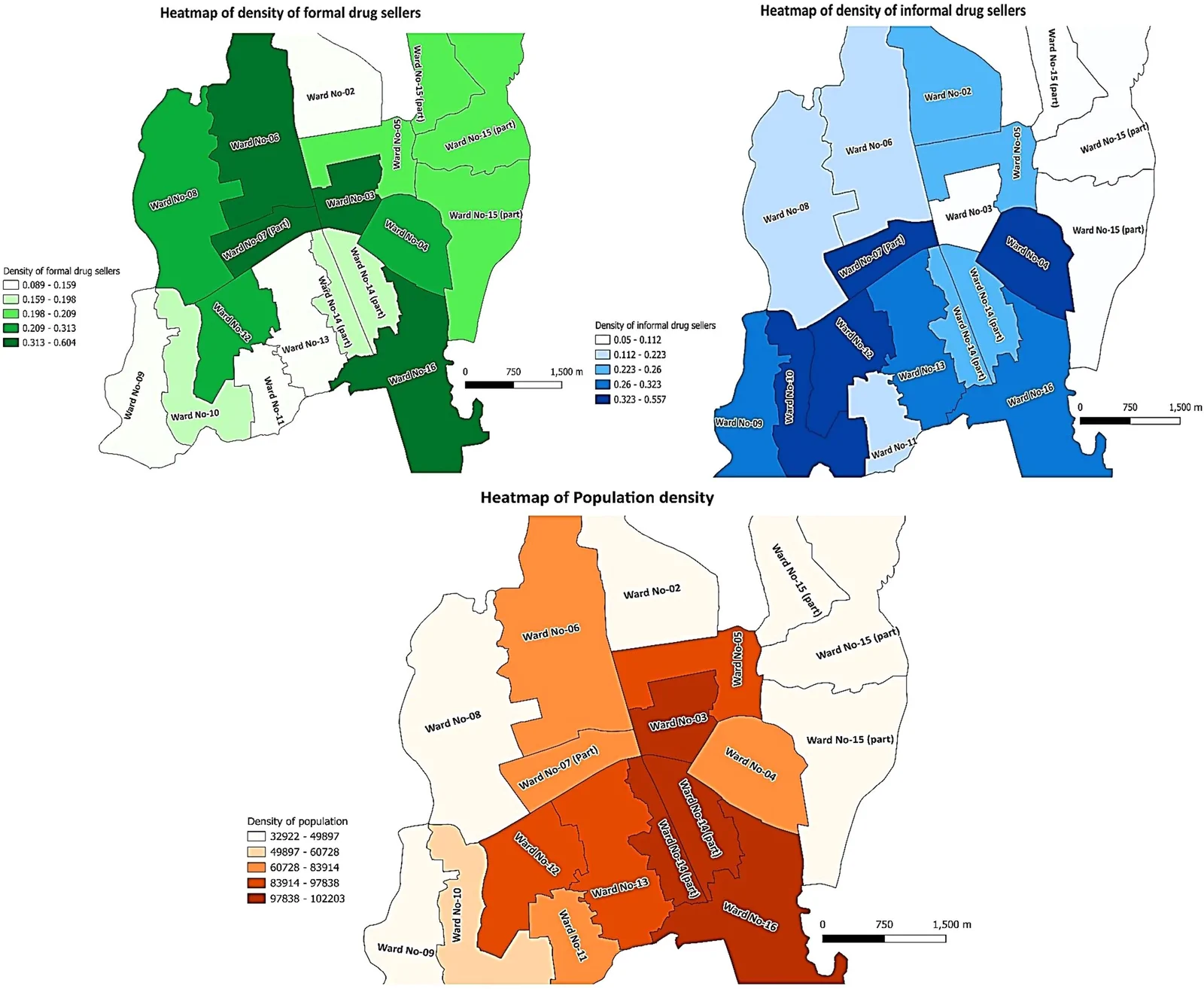
Heat map of formal and informal drug sellers and population density in different wards of Mirpur.

The results showed that drug sellers embedded within densely populated wards of Dhaka provided continuous access to medicines. Of the 1100 drug sellers mapped, 49.9% (*n* = 549) are IHPs (i.e. non-licensed or unregistered providers). The heat map (Fig. 2) of the densities of formal and informal drug sellers per 1000 population in the different wards of Mirpur showed that, apart from Wards 3, 16, 6, and 7 (part) where there is a high presence of formal drug sellers, the informal health providers are more evenly spread across both highly populated wards such as Ward 12 and middle- or less-densely populated wards, including Wards 4, 7, and 10 (Fig. 2). These informal health providers cluster near entry points, bus stands, and local bazaars—areas with high foot traffic and economic activity where there are hundreds of thousands of low-income households. The spatial logic reflects both demand-side constraints (affordability and proximity) and supply-side adaptability (low overhead and flexible hours).

#### Nigeria

**Figure 3:**
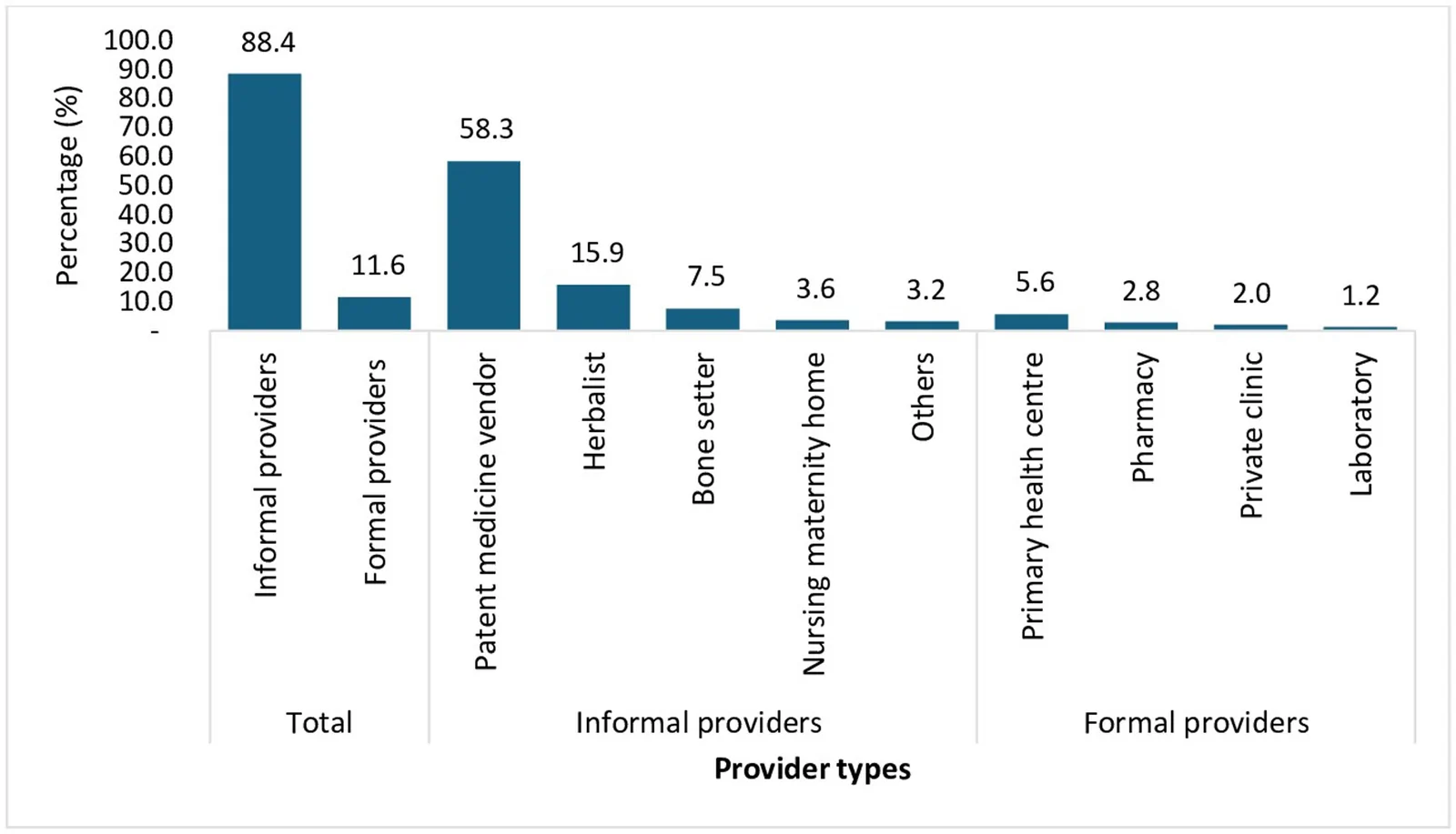
Proportion of formal versus informal providers in eight urban slums in Enugu and Anambra.

The results revealed a dense mix of formal and informal providers in urban slums. Across the eight slums in Enugu and Anambra states considered for the study, the results showed that 88% (*n* = 225) of the providers were IHPs while only 12% (*n* = 30) were formal providers (Fig. 3). These IHPs include PMVs 58.3% (*n* = 149), herbalists 15.9% (*n* = 41), and bone setters 7.5% (*n* = 19), while the formal providers were primary health centres 5.6% (*n* = 14), pharmacies 2.8% (*n* = 7), and private clinics 2.0% (*n* = 5).

#### Nepal

In contrast to other contexts, in Nepal, there was not the preference for informal health providers in urban areas as observed elsewhere. Instead, the results (Table 5) showed that the most preferred health facility by the urban community was government-owned hospitals (55.0%: Ward 4 and 47.1%: Ward 7), followed by private hospitals (30.2%: Ward 7 and 24.3%: Ward 4) and pharmacies (including unregistered pharmacies) and private clinics (6%–8% of responses in both wards). Only a small proportion of households relied on home remedies, ayurvedic centres, or services from female community health volunteers (FCHVs).

**Table 5:** Preferred health facilities by households for care.

Health facilities	Ward 4 (*n* = 2927)	Ward 7 (*n* = 1733)
	*n* (%)	*n* (%)
Never visited a health facility	49 (1.7)	25 (1.4)
Government hospital	1603 (55.0)	816 (47.1)
PHC/primary hospital	18 (0.6)	13 (0.8)
Health post	124 (4.3)	32 (1.8)
Basic healthcare centre	1 (0.05)	40 (2.3)
Community health unit	2 (0.1)	2 (0.1)
Urban health centre	1 (0.05)	2 (0.1)
FCHVs	–	1 (0.1)
Private hospital	709 (24.3)	523 (30.2)
Private clinic	214 (7.3)	132 (7.6)
Pharmacy	177 (6.1)	134 (7.7)
NGO hospital	10 (0.3)	1 (0.1)
NGO clinic	1 (0.05)	6 (0.3)
Home remedy	2 (0.1)	5 (0.3)
Ayurvedic centre	3 (0.1)	1 (0.1)

NGO, non-governmental organization.

A similar result was found when considering disease-specific care access, where the results (Table 6) showed that pharmacies (including unregistered pharmacies) were the most frequently used source of care for communicable diseases (34.8%: Ward 7 and 28.7%: Ward 4), followed by private hospitals (23.8%: Ward 4 and 19.3%: Ward 7). For NCDs, the results revealed government hospitals as the main sources of care in both wards (56.7%: Ward 4 and 45.7%: Ward 7), followed by private hospitals (33.7%: Ward 4 and 44.5%: Ward 7). Home remedies, ayurvedic centres, and treatment abroad were reported only occasionally for both disease categories.

**Table 6:** Type of health facilities and disease-specific care access.

Health facilities	Communicable disease		Non-communicable disease	
	Ward 4 (*n* = 223)	Ward 7 (*n* = 139)	Ward 4 (*n* = 1794)	Ward 7 (*n* = 1107)
	*n* (%)	*n* (%)	*n* (%)	*n* (%)
Government hospital	36 (16.1)	25 (18.5)	1402 (56.7)	701 (45.7)
PHC/primary hospital	3 (1.3)	1 (0.7)	11 (0.4)	19 (1.2)
Health post	12 (5.4)	5 (3.7)	13 (0.5)	6 (0.4)
Urban health centre	–	–	4 (0.2)	2 (0.1)
Private hospital	53 (23.8)	26 (19.3)	833 (33.7)	684 (44.5)
Private clinic	51 (22.8)	27 (20.0)	178 (7.2)	90 (5.9)
Pharmacy	64 (28.7)	47 (34.8)	17 (0.7)	19 (1.2)
Home remedy	4 (1.8)	4 (3.0)	4 (0.2)	2 (0.1)
Ayurvedic centre	–	–	4 (0.2)	4 (0.3)
Aboard treatment	–	–	4 (0.2)	5 (0.3)

#### Ghana

**Figure 4:**
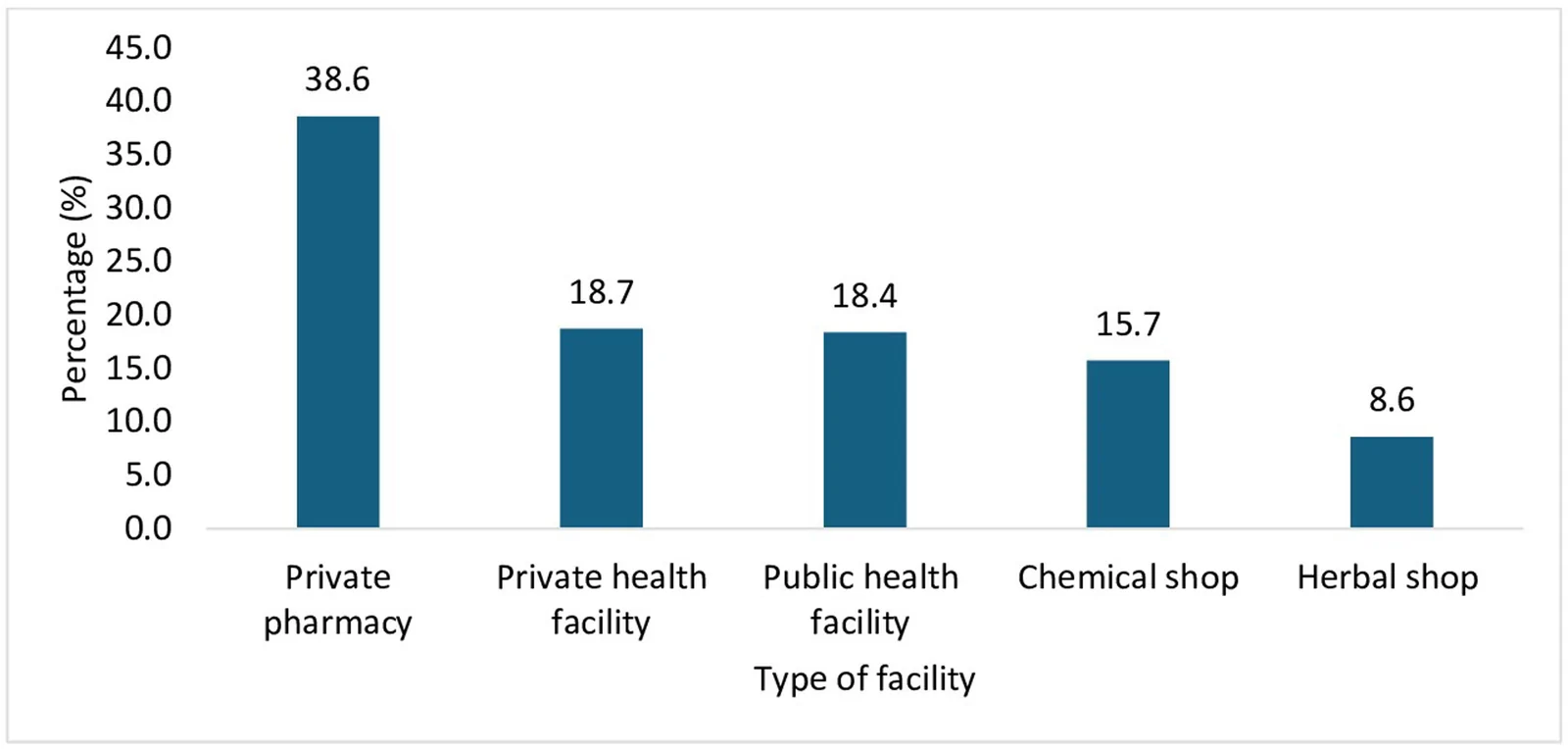
Types of health facilities in the municipality.

In Ghana, the results showed a plurality of healthcare providers co-existed in urban areas, including private health facilities (private clinics, hospitals, and maternity homes), public health facilities (polyclinics, health centre, and Community-based Health Planning and Services zones), private and community pharmacies, chemical shops (drug stores/OTCMS), and herbal shops/facilities. Out of the 267 health facilities surveyed, 38.6% were private pharmacies, while 15.7% and 8.6% were chemical shops and herbal shops/facilities, respectively (Fig. 4).

The results (Table 7) showed that 46% (42%) of diabetes (hypertension) screening services in the municipality are provided by pharmacies. Also, 13.5% and 15% of these services, respectively, are provided by chemical shops. Additionally, a notable percentage, 8.5% and 9% of the diabetes and hypertension management services, respectively, are provided by herbal shops in the municipality. Though a larger proportion (48%–58%) of diabetes, hypertension, and chronic respiratory disease management services are provided by pharmacies and chemical shops, these are medical management services and are due to the medications they sell.

**Table 7:** NCD services provided by health facilities in the municipality.

Health facility type	NCD services				
	Diabetes screening *n* (%)	Diabetes management *n* (%)	Hypertension screening *n* (%)	Hypertension management *n* (%)	Chronic respiratory disease management *n* (%)
Public HF	8 (5.67)	9 (5.88)	25 (15.06)	14 (9.03)	3 (2.63)
Chemical shop	19 (13.48)	13 (8.50)	25 (15.06)	14 (9.03)	10 (8.77)
Herbal shop	4 (2.84)	13 (8.50)	4 (2.41)	14 (9.03)	4 (3.51)
Private HF	44 (31.21)	38 (24.84)	42 (25.30)	39 (25.16)	31 (27.19)
Pharmacy	66 (46.81)	80 (52.29)	70 (42.17)	74 (47.74)	66 (57.89)
Total	141 (100)	153 (100)	166 (100)	155 (100)	114 (100)

HF, health facility.

Similarly, the results (Table 8) showed that pharmacies and chemical shops provide the largest proportions, 50% and 18%, respectively, of malaria diagnosis services compared with the public and private health facilities (5.7% and 31.2%, respectively). In addition, a significant proportion, 9% and 12%, of malaria and sexually transmitted infection (STI) treatment services, respectively, are provided by herbal shops/facilities in the municipality.

**Table 8:** Communicable disease services provided by health facilities in the municipality.

Health facility type	Communicable disease services							
	Malaria diagnosis *n* (%)	Malaria treatment *n* (%)	HIV testing *n* (%)	ART follow-up *n* (%)	STI diagnosis *n* (%)	STI treatment *n* (%)	TB diagnosis *n* (%)	TB treatment *n* (%)
Public HF	12 (6.4)	6 (3.0)	13 (17.6)	8 (22.9)	8 (10.7)	3 (2.3)	3 (12.5)	3 (13.0)
Chemical shop	33 (17.5)	42 (20.8)	1 (1.4)	1 (2.9)	2 (2.7)	10 (7.7)	0 (0.0)	1 (4.4)
Herbal shop	5 (2.7)	19 (9.4)	3 (4.1)	2 (5.7)	3 (4.0)	16 (12.3)	0 (0.0)	1 (4.4)
Private HF	44 (23.3)	38 (18.2)	40 (54.1)	11 (31.4)	43 (57.3)	35 (26.9)	18 (75.0)	15 (65.2)
Pharmacy	95 (50.3)	97 (48.0)	17 (23.0)	13 (37.1)	19 (25.3)	66 (50.8)	3 (12.5)	3 (13.0)
Total	189 (100.0)	202 (100.0)	267 (100.0)	35 (100.0)	75 (100.0)	130 (100.0)	24 (100.0)	23 (100.0)

ART, antiretroviral therapy; HF, health facility; HIV, human immunodeficiency virus.

#### Factors that determine where healthcare is sought by respondents in Ghana

**Figure 5:**
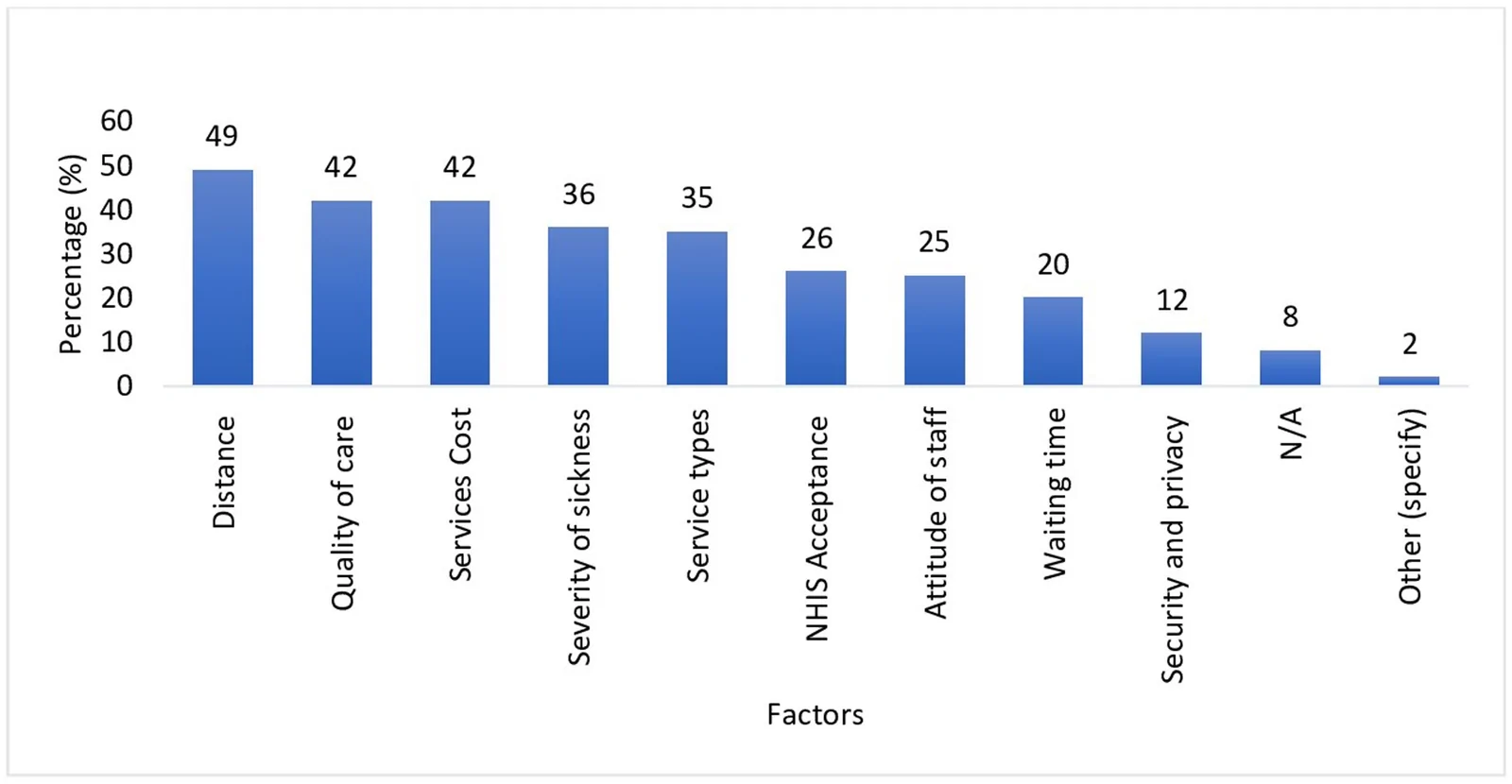
Factors that determine where healthcare is sought by respondents.

The results from the household survey showed that the choice of where community members seek healthcare is largely influenced by the physical distance (49%), perceived quality of the care (42%), cost of care (42%), severity of the ailment (36%), and types of primary care service provided by the facilities (35%; Fig. 5).

## Discussion

We explored the role of the informal healthcare providers within urban contexts of Bangladesh, Nepal, Nigeria, and Ghana to enhance understanding of their potential within health systems and to promote their integration into the mainstream health system to improve access and quality of care for the urban poor.

Our findings showed that across the four countries, there is substantial evidence that the IHPs make significant contributions to health service delivery by bridging the care gap, particularly for socially and economically disadvantaged populations, serving as the first points of contact by providing an extensive range of health services, including preventive, curative, and restorative services. However, IHPs are loosely defined and lack routine monitoring by regulatory bodies, leading to massive growth of the sector without robust standards, accreditation, and protocols.

These findings are consistent with earlier reports in the literature (Adams et al. 2015, Mackintosh et al. 2016, Kumah 2022). Our analyses revealed that the growth of the informal sector is often driven by rapid migration caused by unplanned urbanization, which outpaces the development of formal health infrastructure and creates demand for informal care, a mismatch between public resources and healthcare needs, and the proximity of the informal care to those in need. In Nepal, a major factor influencing the choice and use of health facilities is household wealth quintile and the number of years of schooling an individual has (Rahman 2022). Across the countries, it has also been revealed that urban health policies have historically neglected informal providers. Studies elsewhere have also shown that, belief systems of the urban poor play a significant role in informal healthcare utilization (Conteh et al. 2025). For example, an earlier study in an informal settlement in Freetown, Sierra Leone, found that people often seek care from biomedical, traditional, and religious providers simultaneously. Their choices are based on their understanding of their conditions and their belief systems, where they switch between public, private, and traditional options depending on the perceived effectiveness of treatment and the worsening of their conditions (Conteh et al. 2025).

In Nepal, we observed less utilization of the IHPs, as they are largely concentrated in rural settings. Although the National Health Policy 2019 and the Nepal Health Sector Strategy (2016–2021) have prioritized the establishment of rural health posts and basic health facilities, these disparities still persist. The government-led Nepal Health Facility Surveys (2021 and 2025) show a higher concentration of advanced facilities and human resources in urban municipalities compared with rural areas (Ministry of Health and Population 2021). In contrast, urban settings have a wide range of formal healthcare facilities, including public and private hospitals, clinics, and licensed pharmacies. Consequently, urban populations primarily seek care from formal health facilities, whereas rural populations often rely on informal and unregistered providers (Ban et al. 2020). Urban residents in Nepal also tend to have higher literacy, greater health awareness, and better access to diverse and formal private-sector services, supported by improved education, infrastructure, and resources (Saito et al. 2016). This pattern aligns with broader evidence from South Asia, which shows that informal providers often emerge in settings with limited access to trained human resources and weak health infrastructure (Sudhinaraset et al. 2013).

The lack of robust standards, regulation, accreditation, supervision, and treatment protocols, despite the extensive provision of health services, raises concerns about the quality of care in the informal sector. In Nepal, it is estimated that ∼3000 unregistered pharmacies are operating across the country (Ministry of Health and Population [Nepal] New ERA 2023). In both Nepal and Bangladesh, routine monitoring of the private healthcare providers, including IHPs, by regulatory bodies is limited (Gurung and Gauld 2016, Ministry of Health and Family Welfare Bangladesh 2022). Similarly, in Ghana, the Health Facility Regulatory Agency is tasked with licensing, monitoring, and regulating both public and private health facilities, but this mandate does not cover the informal sector. Furthermore, while drug stores/chemical sellers are licensed to dispense over-the-counter medicines, most practitioners lack formal training, resulting in limited knowledge of appropriate medical practices and treatment guidelines for various illnesses.

In other LMICs, there is abundant evidence that only a few informal providers have qualified personnel (Okechi et al. 2025), while the majority offer services without proper training, supervision, regulation, or knowledge of risk factors (Iqbal et al. 2019). In India, Zambia, and Congo, a significant portion of antibiotic dispensing is attributed to informal health providers, contributing to the global challenge of antimicrobial resistance due to inappropriate and poor prescribing practices (Nahar et al. 2020, Ingelbeen et al. 2022, Thapa et al. 2022, Wildbret et al. 2023).

These findings underscore the urgent need to prioritize, engage, or integrate IHPs into the formal health system to address issues of access to services, whilst also improving quality through regulation and monitoring. A crucial aspect of health system stewardship is the effective regulation of the private sector to implement legal provisions and ensure adherence to national standards, thereby promoting quality care and protecting consumers’ rights. Recent studies (Lilford et al. 2025) have underscored the necessity to enhance regulation and management to shape the PHC economy in urban areas. To successfully and effectively link IHPs to the formal health system, it is essential to understand the prevailing contextual factors and how they can be adjusted to support these connections (Onwujekwe et al. 2025b). Onwujekwe et al. (2025b) highlight the importance of individual, organizational, and external factors that either hinder or promote effective linkage. Individual factors include personal attitudes towards linkage, the capability of informal providers to deliver quality services, the nature of existing relationships between formal and informal providers, and trust in the formal health system. Organizational factors consist of leadership structure, coordination and accountability mechanisms, the functional management capacity of the formal health system, and multiple regulatory frameworks. External factors involve supportive health policies on integration, sustainable funding for continuous training and supervision, and global support for integration (Onwujekwe et al. 2025b).

Linking IHPs to the formal health system has proven effective in regulation and significantly improved access to and the quality of health services. Such linkages are often seen as a win–win situation for the health sector, resulting in improved health status of urban dwellers, particularly the poor and vulnerable populations (Onwujekwe et al. 2025b). In Zambia, when TBAs received training, they were found to provide counselling, referrals, and logistical support, including treatment adherence assistance, to formal care providers (Muzyamba et al. 2017). Similarly, in Pakistan, TBAs support community health midwives in normal deliveries and refer high-risk cases to the formal health system (Shaikh et al. 2014). Furthermore, the experience from Ghana illustrates how herbal medicine can be incorporated into the formal system (Asase 2023). This integration offers a means to regulate and harness the sector, thereby increasing access for those in underserved urban areas. There is a clear interest among informal providers to enhance this integration (Onwujekwe et al. 2022, 2025a, 2025b); however, comprehensive system-wide solutions are necessary.

The study has some limitations. First, as it is common with rapid literature reviews (Speckemeier et al. 2022, Stevens et al. 2025), the literature search was not as methodologically rigorous as that for a systematic review. We also did not quantify the descriptive summaries of the review because of the nature of the evidence we gathered. Despite these limitations, the available evidence retrieved was able to provide an answer to the review questions. Also, the case studies adopted descriptive approaches, which could not directly establish a statistically significant association between informal primary care providers and contextual factors that influence their utilization or expansion, as this additional information was beyond the scope of this study. The strength of the study is the multiple data sources and countries illustrating comprehensive evidence on the role of IHPs in improving access to care.

## Conclusion

In many, but not all LMIC urban contexts, IHPs are filling the gap left by formal providers by delivering extensive healthcare services, especially to the socially and economically disadvantaged in urban areas. They offer potential for enhancing access to care and can become a gateway to primary and higher levels of care. However, there are concerns regarding the quality of care, as the informal sector lacks trained health professionals, robust standards, accreditation, and regulations. The study underscores the need to improve healthcare access and quality for the urban poor and linking the informal sector with the formal sector or directly integrating informal providers into the country's mainstream health system present a viable approach with the potential to accelerate LMICs’ progress towards UHC.

## Supplementary Material

czag087_Supplementary_Data

## Data Availability

The data generated or analysed for this study are included in this published article.

## Reflexivity statement

The group of authors included eight females and nine males, comprising researchers based in Bangladesh, Ghana, Nepal, Nigeria, and the UK, with multiple levels of seniority from predoctoral researchers to postdoctoral research fellows, associate and full professors, exemplifying inclusivity. All authors have extensive experience in health systems and policy research and brought country context-specific insights in shaping the paper. The outsider perspectives of the UK team who are not from the countries where the research was conducted brought valuable, unbiased insights that significantly enriched the analysis, resulting in a more impactful and credible paper.

## References

[czag087-B1] Abimbola S, Ogunsina K, Charles-Okoli AN et al Information, regulation and coordination: realist analysis of the efforts of community health committees to limit informal health care providers in Nigeria. Health Econ Rev 2016;6:. 10.1186/s13561-016-0131-5 PMC5108730 27844451

[czag087-B2] Adams AM, Islam R, Ahmed T. Who serves the urban poor? A geospatial and descriptive analysis of health services in slum settlements in Dhaka, Bangladesh. Health Policy Plan 2015;30:i32–45. 10.1093/heapol/czu094 PMC4353891 25759453

[czag087-B3] Adams AM, Nambiar D, Siddiqi S et al Advancing universal health coverage in South Asian cities: a framework. BMJ (Online) 2018;363:k4905. 10.1136/bmj.k4905 PMC7115914 30498010

[czag087-B4] Ahmed SM, Hossain A, Rajachowdhury AM et al The health workforce crisis in Bangladesh: shortage, inappropriate skill-mix and inequitable distribution. Hum Resour Health 2011;9:3. 10.1186/1478-4491-9-3 PMC3037300 21255446

[czag087-B5] Arize I, Ozughalu J, Okechi B et al Assessing informal healthcare providers’ knowledge of diagnosis and treatment of malaria and diarrhea: evidence from urban informal settlements in Southeast Nigeria. Front Public Health 2025;13:1556996. 10.3389/fpubh.2025.1556996 PMC11937094 40145002

[czag087-B6] Arteaga F, Ferrer-Riquelme AJ. Missing data. In: Brown SD, Tauler R, Walczak B (eds.), Comprehensive chemometrics chemical and biochemical data analysis. Amsterdam: Elsevier, 2009, 285–314.

[czag087-B7] Asase A . Ghana’s herbal medicine industry: prospects, challenges and ways forward from a developing country perspective. Front Pharmacol 2023;14:1267398. 10.3389/fphar.2023.1267398 PMC10579918 37854720

[czag087-B8] Asiedu SA, Hulscher M, Abdulai MA et al Stakeholders’ perspectives on training over the counter medicine sellers and community-based Health Planning and Services facilities to dispense antibiotics in Ghana. J Pharm Policy Pract 2021;14:62. 10.1186/s40545-021-00349-0 PMC8299568 34294159

[czag087-B9] Ban B, Ban B, Hodgins S et al A national survey of private-sector outpatient care of sick infants and young children in Nepal. BMC Health Serv Res 2020;20:545–16. 10.1186/s12913-020-05393-1 PMC7298835 32546276

[czag087-B10] Bangladesh Bureau of Statistics . Population and Housing Census 2022: Community Report. Dhaka, Bangladesh: Statistics and Informatics Division, Ministry of Planning, 2025.

[czag087-B11] Basabih M, Prasojo E, Rahayu AYS. Hospital services under public–private partnerships, outcomes and, challenges: a literature review. J Public Health Res 2022;11:22799036221115781. 10.1177/22799036221115781 PMC9424887 36052099

[czag087-B12] Bloom G, Standing H. Pluralism and Marketisation in the Health Sector: Meeting Health Needs in Contexts of Social Change in low and Middle-Income Countries. England: Institute of Development Studies, 2001.

[czag087-B13] Bloom G, Standing H, Lloyd R. Markets, information asymmetry and health care: towards new social contracts. Soc Sci Med 2008;66:2076–87. 10.1016/j.socscimed.2008.01.034 18316147

[czag087-B14] Bloom G, Standing H, Lucas H et al Making health markets work better for poor people: the case of informal providers. Health Policy Plan 2011;26:i45–52. 10.1093/heapol/czr025 21729917

[czag087-B15] Bradley S, Lauren R, Tess S. Sources for Sick Child Care in 24 USAID Priority Countries. Brief. Rockville, MD: Sustaining Health Outcomes Through the Private Sector Plus Project. Bethesda: Abt Associates, 2018.

[czag087-B16] Brieger W, Salako L, Umeh R et al Promoting prepackaged drugs for prompt and appropriate treatment of febrile illnesses in rural Nigerian communities. Int Q Community Health Educ 2002;21:19–40. 10.2190/0N5X-0NVD-R0VB-4QKF

[czag087-B17] Clarke D, Doerr S, Hunter M et al The private sector and universal health coverage. Bull World Health Organ 2019;97:434–5. 10.2471/BLT.18.225540 PMC6560377 31210681

[czag087-B18] Clay B . Barriers to the safe preservation of the traditional herbal practice in Ghana. World Med Health Pol 2024;16:506–13. 10.1002/wmh3.612

[czag087-B19] Conteh A, Dean L, Wilkinson A et al Health seeking by people living with non-communicable diseases in a pluralistic health system: the role of informal healthcare providers. Int J Equity Health 2025;24:67. 10.1186/s12939-025-02428-z PMC11895327 40069705

[czag087-B20] de Siqueira Filha N, Li J, Phillips-Howard P et al The economics of healthcare access: a scoping review on the economic impact of healthcare access for vulnerable urban populations in low- and middle-income countries. Int J Equity Health 2022;21:191. 10.1186/s12939-022-01804-3 PMC9805259 36585704

[czag087-B21] Elsey H, Agyepong I, Huque R et al Rethinking health systems in the context of urbanisation: challenges from four rapidly urbanising low-income and middle-income countries. BMJ Glob Health 2019;4:e001501. 10.1136/bmjgh-2019-001501 PMC6577312 31297245

[czag087-B22] Ezeh A, Oyebode O, Satterthwaite D et al The history, geography, and sociology of slums and the health problems of people who live in slums. Lancet 2017;389:547–58. 10.1016/S0140-6736(16)31650-6 27760703

[czag087-B23] Fayehun O, Apenteng P, Umar UA et al Diagnosis of cancer in the South and North of Nigeria: duration and causes of delay. BMC Health Serv Res 2025;25:738. 10.1186/s12913-025-12707-8 PMC12093698 40399840

[czag087-B24] Frempong BK, Amalba A, Donkor N et al Regulatory compliance among over-the-counter medicine sellers facilities within the Upper East Region of Ghana. J Pharm Policy Pract 2021;14:72. 10.1186/s40545-021-00363-2 PMC8383477 34429161

[czag087-B25] GBC Ghana . Herbal medicine practice integrated into healthcare delivery system. gbcghanaonline.com, 2019.

[czag087-B26] Ghana Statistical Service . Ghana Demographic and Health Survey 2022. Accra: Ghana Statistical Service, 2022.

[czag087-B27] Grant MJ, Booth A, Centre S. A typology of reviews: an analysis of 14 review types and associated methodologies. Health Info Libr J 2009;26:91–108. 10.1111/j.1471-1842.2009.00848.x 19490148

[czag087-B28] Gurung G, Gauld R. Private gain, public pain: does a booming private healthcare industry in Nepal benefit its people?. BMJ 2016. https://blogs.bmj.com/bmj/2016/09/30/does-abooming-privatehealthcare-industry-in-nepal-benefit-itspeople (Retrieved 20 April 2025).

[czag087-B29] Hampshire K, Mariwah S, Amoako-sakyi D et al It is very difficult in this business if you want to have a good conscience”: pharmaceutical governance and on-the-ground ethical labour in Ghana. Glob Bioeth 2022;33:103–21. 10.1080/11287462.2022.2103899 PMC9331207 35912379

[czag087-B100] Heijden J, Gray N, Stringer B, et al ‘Working to stay healthy', health-seeking behaviour in Bangladesh’s urban slums: a qualitative study. BMC Public Health 2019;19:600. 10.1186/s12889-019-6750-0 PMC6525448 31101099

[czag087-B30] HERD International . Health Facility Assessment of Pokhara Metropolitan City of Nepal—2022 Summary Report. Lalitpur: HERD International, 2023.

[czag087-B31] Hossain MR, Georgi NW, Noor FM et al Healthcare in the margins: a qualitative study of healthcare access and utilization in Bangladesh’s Informal Urban Settlements. J Urban Health 2025;102:1298–309. 10.1007/s11524-025-01042-2 PMC12738492 41413684

[czag087-B32] Ingelbeen B, Phanzu D, Phoba M-F et al Antibiotic use from formal and informal Healthcare providers in the Democratic Republic of Congo: a population-based study in two health zones. Clin Microbiol Infect 2022;28:1272–7. 10.1016/j.cmi.2022.04.002 35447342

[czag087-B33] Iqbal MZ, Khan AH, Iqbal MS et al Review of pharmacist-led interventions on diabetes outcomes: an observational analysis to explore diabetes care opportunities for pharmacists. J Pharm Bioallied Sci 2019;11:299–309. 10.4103/jpbs.JPBS_138_19 PMC6791080 31619911

[czag087-B34] Jan S, Laba TL, Essue BM et al Action to address the household economic burden of NCDs. Lancet 2018;391:2047–58. 10.1016/S0140-6736(18)30323-4 29627161

[czag087-B35] Kohrt BA, Harper I. Navigating diagnoses: understanding mind–body relations, mental health, and stigma in Nepal. Cult Med Psychiatry 2008;32:462–91. 10.1007/s11013-008-9110-6 PMC3869091 18784989

[czag087-B36] Kruk ME, Gage AD, Arsenault C et al High-quality health systems in the sustainable development goals era: time for a revolution. Lancet Glob Health Comm 2018;6:e1196–252. 10.1016/S2214-109X(18)30386-3 PMC7734391 30196093

[czag087-B37] Kumah E . The informal healthcare providers and universal health coverage in low and middle-income countries. Glob Health 2022;18:45. 10.1186/s12992-022-00839-z PMC9044596 35477581

[czag087-B38] Kunwar RM, Shrestha KP, Bussmann RW. Traditional herbal medicine in far-west Nepal : a pharmacological appraisal. J Ethnobiol Ethnomed 2010;6:35. 10.1186/1746-4269-6-35 PMC3012020 21144003

[czag087-B39] Lilford RJ, Daniels B, McPake B et al Policy and service delivery proposals to improve primary care services in low-income and middle-income country cities. Lancet Glob Health 2025;13:e954–66. 10.1016/S2214-109X(24)00536-9 40288403

[czag087-B40] Mackintosh M, Channon A, Karan A et al What is the private sector? Understanding private provision in the health systems of low-income and middle-income countries. Lancet (London, England) 2016;388:596–60. 10.1016/S0140-6736(16)00342-1 27358253

[czag087-B41] Mbachu C, Arize I, Obi C et al Assessing knowledge of hypertension and diabetes mellitus among informal healthcare providers in urban slums in southeastern Nigeria. Discov Public Health 2024;21:21. 10.1186/s12982-024-00143-8

[czag087-B42] Miller R, Goodman C. Performance of retail pharmacies in low- and middle-income Asian settingss: a systematic review. Health Policy Plan 2016;31:940–95. 10.1093/heapol/czw007 PMC4977427 26962123

[czag087-B43] Ministry of Health and Family Welfare Bangladesh . Strategic Investment Plan of the 5th Health, Nutrition and Population Sector Programme. Dhaka, Bangladesh: Planning Wing, Ministry of Health and Family Welfare, 2022.

[czag087-B44] Ministry of Health and Population . Nepal Health Facility Survey. Kathmandu, Nepal: Ministry of Health and Population, 2021.

[czag087-B45] Ministry of Health and Population [Nepal], New ERA . Nepal Demographic and Health Survey 2022. Kathmandu, Nepal: Ministry of Health and Population, 2023.

[czag087-B46] Mundia C, Murayama Y. Modeling spatial processes of urban growth in African cities: a case study of Nairobi City. Urban Geogr 2010;31:259–72. 10.2747/0272-3638.31.2.259

[czag087-B47] Muzyamba C, Groot W, Tomini S et al The usefulness of traditional birth attendants to women living with HIV in resource-poor settings: the case of Mfuwe, Zambia. Trop Med Health 2017;45:37. 10.1186/s41182-017-0076-3 PMC5683231 29158723

[czag087-B48] Nahar P, Unicomb L, Lucas P et al What contributes to inappropriate antibiotic dispensing among qualified and unqualified Healthcare providers in Bangladesh? A qualitative study. BMC Health Serv Res 2020;20:656. 10.1186/s12913-020-05512-y PMC7362537 32669092

[czag087-B49] Nepal A, Hendrie D, Robinson S et al Survey of the pattern of antibiotic dispensing in private pharmacies in Nepal. BMJ Open 2019;9:e032422. 10.1136/bmjopen-2019-032422 PMC6797298 31601603

[czag087-B50] Obi C, Arize I, Nwokolo C et al Referral experiences of healthcare consumers: results from a cross-sectional study in urban slums in southeast Nigeria. Front Public Health 2025;13:1561158. 10.3389/fpubh.2025.1561158 PMC12149103 40496460

[czag087-B51] Odii A, Arize I, Agwu P et al To what extent are informal healthcare providers in slums linked to the formal health system in providing services in sub-Saharan Africa? A 12-year scoping review. J Urban Health 2024;101:1248–58. 10.1007/s11524-024-00885-5 PMC11652447 38874863

[czag087-B52] Okechi B, Orjiakor CT, Nwokolo C et al Community leaders’ perspectives on linking formal and informal health providers in Nigerian urban slums: a qualitative study. Discov Soc Sci Health 2025;5:61. 10.1007/s44155-025-00205-5 PMC12045822 40322050

[czag087-B53] Onuh P, Agwu P, Mbachu C et al Informal–formal healthcare services delivery nexus in Nigeria’s urban slums: a reconnaissance study. J Soc Serv Res 2024;50:711–24. 10.1080/01488376.2024.2371859

[czag087-B54] Onwujekwe O, Ezema GU, Nwokolo C et al Willingness to pay for improved quality of services from informal health providers in urban slums in Nigeria. PharmacoEconomics - Open 2025a;9:917–29. 10.1007/s41669-025-00597-9 PMC12559521 40864217

[czag087-B55] Onwujekwe O, Hanson K, Uzochukwu B. Do poor people use poor quality providers? Evidence from the treatment of presumptive malaria in Nigeria. Trop Med Int Health 2011;16:1087–98. 10.1111/j.1365-3156.2011.02821.x 21702870

[czag087-B56] Onwujekwe O, Mbachu CO, Agyepong I et al Institutionalizing linkages between informal healthcare providers and the formal health system in Nigeria: what are the facilitating and constraining contextual influences? Health Policy Plan 2025b;40:471–82. 10.1093/heapol/czaf009 PMC11979586 39913199

[czag087-B57] Onwujekwe O, Orjiakor CT, Odii A et al Examining the roles of stakeholders and evidence in policymaking for inclusive urban development in Nigeria: findings from a policy analysis. Urban Forum 2022;33:505–53. 10.1007/s12132-021-09453-5

[czag087-B58] Ozor O, Nwokolo C, Teixeira de Siqueira Filha N et al Inequities in household out-of-pocket spending among urban slum dwellers in Southeast Nigeria. Int J Public Health 2025;70:1607969. 10.3389/ijph.2025.1607969 PMC11981907 40212065

[czag087-B59] Page M, McKenzie J, Bossuyt P et al The PRISMA 2020 statement: an updated guideline for reporting systematic reviews. BMJ 2021;372:n71. 10.1136/bmj.n71 PMC8005924 33782057

[czag087-B60] Penchansky R, Thomas JW. The concept of access: definition and relationship to consumer satisfaction. Med Care 1981;19:127–40. 10.1097/00005650-198102000-00001 7206846

[czag087-B61] Rahman R . Private sector healthcare in Bangladesh: implications for social justice and the right to healthcare. Glob Public Health 2022;17:285–96. 10.1080/17441692.2020.1858136 33301702

[czag087-B62] Saito E, Gilmour S, Yoneoka D et al Inequality and inequity in healthcare utilization in urban Nepal: a cross-sectional observational study. Health Policy Plan 2016;31:817–24. 10.1093/heapol/czv137 PMC4977425 26856362

[czag087-B63] Scott-Emuakpor A . The evolution of health care systems in Nigeria: which way forward in the twenty-first century. Niger Med J 2013;51:53–65. 10.4314/nmj.v51i2.59866

[czag087-B64] Shaikh B, Khan S, Maab A et al Emerging role of traditional birth attendants in mountainous terrain: a qualitative exploratory study from Chitral District, Pakistan. BMJ Open 2014;4:e006238. 10.1136/bmjopen-2014-006238 PMC4248099 25428631

[czag087-B65] Sieverding M, Beyeler N. Integrating informal providers into a people-centered health systems approach: qualitative evidence from local health systems in rural Nigeria. BMC Health Serv Res 2016;16:526. 10.1186/s12913-016-1780-0 PMC5041446 27687854

[czag087-B66] Sizear M, Nababan H, Siddique M et al Perceptions of appropriate treatment among the informal allopathic providers: insights from a qualitative study in two peri-urban areas in Bangladesh. BMC Health Serv Res 2019;19:424. 10.1186/s12913-019-4254-3 PMC6595608 31242900

[czag087-B67] Speckemeier C, Niemann A, Wasem J et al Methodological guidance for rapid reviews in healthcare: a scoping review. Res Synth Methods 2022;13:394–404. 10.1002/jrsm.1555 35247034

[czag087-B68] Stevens A, Hersi M, Garritty C et al Rapid review method series: interim guidance for the reporting of rapid reviews. BMJ Evid Based Med 2025;30:118–23. 10.1136/bmjebm-2024-112899 PMC12013547 39038926

[czag087-B69] Subedi B . Legitimising Traditional Healers. The Kathmandu Post, 2023a. https://kathmandupost.com/columns/2023/04/27/legitimisingtraditional-healers (Retrieved June 2025).

[czag087-B70] Subedi B . Perspective chapter: integrating traditional healers into the national health care system—a review and reflection. In: Christian R (ed.), ed.Rural Health—Investment, Research and Implications. London: IntechOpen, 2023b, 1–16.

[czag087-B71] Sudhinaraset M, Ingram M, Lofthouse HK et al What is the role of informal healthcare providers in developing countries? A systematic review. PLoS One 2013;8:e54978. 10.1371/journal.pone.0054978 PMC3566158 23405101

[czag087-B72] Tandan M, Thapa P, Bhandari B et al Antibiotic dispensing practices among informal healthcare providers in low­income and middle-income countries: a scoping review protocol. BMJ Open 2024;14:e086164. 10.1136/bmjopen-2024-086164 PMC11191789 38904128

[czag087-B73] Tenkorang EY . Health provider characteristics and choice of health care facility among Ghanaian health seekers. Health Syst Reform 2016;2:160–70. 10.1080/23288604.2016.1171282 31514639

[czag087-B74] Thapa P, Hall J, Jayasuriya R et al What are the tuberculosis care practices of informal healthcare providers? A cross-sectional study from eastern India. Health Policy Plan 2022;37:1158–66. 10.1093/heapol/czac062 35920775

[czag087-B75] Thapa P, Jayasuriya R, Hall JJ et al Role of informal healthcare providers in tuberculosis care in low- and middle-income countries: a systematic scoping review. PLoS One 2021;16:e0256795. 10.1371/journal.pone.0256795 PMC8412253 34473752

[czag087-B76] Tiwari G, Thakur A, Pokhrel S et al Health care seeking behavior for common childhood illnesses in Birendranagar municipality, Surkhet, Nepal: 2018. PLoS One 2022;17:e0264676. 10.1371/journal.pone.0264676 PMC8967048 35353836

[czag087-B77] WHO . Bangladesh Health System Review. Geneva: WHO, 2015.

[czag087-B78] WHO . Delivering Quality Health Services: a Global Imperative for Universal Health Coverage. Geneva: WHO, 2018.

[czag087-B79] Wildbret S, Stuck L, Luchen C et al Drivers of informal sector and non-prescription medication use in pediatric populations in a low- and middle-income setting: a prospective cohort study in Zambia. PLOS Glob Public Health 2023;3:e0002072. 10.1371/journal.pgph.0002072 PMC10325117 37410740

[czag087-B80] World Health Organization . Declaration of Astana: Global Conference on Primary Health Care. Astana, Kazakhstan: World Health Organization, 2018. https://www. who.int/publications/i/item/WHO-HIS-SDS-2018.61 (1 December 2024, date last accessed).

[czag087-B81] World Health Organization . Engaging the Private Health Service Delivery Sector Through Governance in Mixed Health Systems: Strategy Report of the WHO Advisory Group on the Governance of the Private Sector for Universal Health Coverage. Geneva: World Health Organization, 2020.

[czag087-B82] World Health Organization . Universal Health Coverage (UHC). Geneva: World Health Organisation, 2023.

[czag087-B83] Yirenkyi A. 2019. The role of traditional medicine in Ghana towards achieving universal health coverage: the journey so far. Ghana News. https://ghananewsonline.com.gh/the-role-of-traditional-medicine-in-ghana-towards-achieving-universal-health-coverage-the-journey-so-far/

