# Supplementary material for: Exploring the roles of informal healthcare providers in urban settings to improve access and quality of care for the urban poor: experience from Bangladesh, Ghana, Nepal, and Nigeria
Source: Health Policy Plan. 2026 Jul 8;41(8):1358–74. doi: 10.1093/heapol/czag087 (PMC13586622; doi:10.1093/heapol/czag087)
Supplement: Supplementary file 1 [file czag087_supplementary_data.zip › Supplementary file 2.docx]

**Final search term and variations**

| **Main term** | **Variations of the term searched** |
| --- | --- |
| Informal healthcare in urban context | Primary healthcare; informal healthcare providers; urban health; quality of care; access to care; informal healthcare definition; evolution; regulation; utilization; beneficiaries; types; roles; governance; services |
| Country context | Bangladesh, Ghana, Nepal, Nigeria |
