# Supplementary material for: Exploring the roles of informal healthcare providers in urban settings to improve access and quality of care for the urban poor: experience from Bangladesh, Ghana, Nepal, and Nigeria
Source: Health Policy Plan. 2026 Jul 8;41(8):1358–74. doi: 10.1093/heapol/czag087 (PMC13586622; doi:10.1093/heapol/czag087)
Supplement: Supplementary file 1 [file czag087_supplementary_data.zip › Supplementary file 3_Documents_studies reviewed.docx]

**Supplementary file 3: Documents and studies reviewed**

| **Document/Study type** | **Authors (Year)** | **Country** | **Title** |
| --- | --- | --- | --- |
| Peer-reviewed article | Abimbola *et al.*, (2016) | Nigeria | Information, regulation and coordination: realist analysis of the efforts of community health committees to limit informal health care providers in Nigeria |
| Peer-reviewed article | Ahmed *et al.*, (2011) | Bangladesh | The health workforce crisis in Bangladesh : shortage, inappropriate skill-mix and inequitable distribution |
|  | Asase, (2023) | Ghana | Ghana’s herbal medicine industry: prospects, challenges and ways forward from a developing country perspective |
| Peer-reviewed article | Asiedu *et al.*, (2021) | Ghana | Stakeholders ’ perspectives on training over-the-counter medicine sellers and Community ‑ based Health Planning and Services facilities to dispense antibiotics in Ghana. |
| Peer-reviewed article | Arize *et al.*, (2025) | Nigeria | Assessing informal healthcare providers’ knowledge of diagnosis and treatment of malaria and diarrhea: evidence from urban informal settlements in Southeast Nigeria |
| Peer-reviewed article | Ban *et al.*, (2020) | Nepal | A national survey of private-sector outpatient care of sick infants and young children in Nepal |
| Peer-reviewed article | Bloom *et al.*, (2011) | Multi-country | Making health markets work better for poor people: The case of informal providers |
| Project report | Bradley *et al.*, (2018) | Nepal | Sources for Sick Child Care in 24 USAID Priority Countries. Brief. Rockville, MD: Sustaining Health Outcomes through the Private Sector Plus Project |
| Peer-reviewed article | Brieger *et al.*, (2002) | Nigeria | Promoting Prepackaged Drugs for Prompt and Appropriate Treatment of Febrile Illnesses in Rural Nigerian Communities |
| Peer-reviewed article | Clay (2024) | Ghana | Barriers to the safe preservation of the traditional herbal practice in Ghana. |
| Peer-reviewed article | Frempong *et al.*, (2021) | Ghana | Regulatory compliance among over ‑ the ‑ counter medicine sellers facilities within the Upper East Region of Ghana |
| Media report | GBC Ghana (2019) | Ghana | Herbal medicine practice integrated into healthcare delivery system |
| National policy document | Ghana Statistical Service (2022) | Ghana | Ghana Demographic and Health Survey 2022. |
| Peer-reviewed article | Hampshire *et al.*, (2022) | Ghana | “It is very difficult in this business if you want to have a good conscience”: pharmaceutical governance and on-the-ground ethical labour in Ghana. |
| Peer-reviewed article | Heijden *et al.*, (2019) | Bangladesh | ‘Working to stay healthy’, health-seeking behaviour in Bangladesh’s urban slums: a qualitative study. |
| Research document | HERD International (2023) | Nepal | Health Facility Assessment of Pokhara Metropolitan City of Nepal -2022 Summary Report. |
| Peer-reviewed article | Hossain *et al.*, (2025) | Bangladesh | Healthcare in the Margins: A Qualitative Study of Healthcare Access and Utilization in Bangladesh’s Informal Urban Settlements. |
| Peer-reviewed article | Kohrt and Harper (2008) | Nepal | Navigating diagnoses: Understanding mind–body relations, mental health, and stigma in Nepal |
| Peer-reviewed article | Kunwar *et al.*, (2010) | Nepal | Traditional herbal medicine in Far-west Nepal : a pharmacological appraisal. |
| Peer-reviewed article | Nahar *et al.*, (2020) | Bangladesh | What contributes to inappropriate antibiotic dispensing among qualified and unqualified Healthcare providers in Bangladesh? A qualitative study. |
| Peer-reviewed article | Nepal *et al.*, (2019) | Nepal | Survey of the pattern of antibiotic dispensing in private pharmacies in Nepal. |
| Peer-reviewed article | Odii *et al.*, (2024) | Nigeria | To What Extent Are Informal Healthcare Providers in Slums Linked to the Formal Health System in Providing Services in Sub-Sahara Africa? A 12-Year Scoping Review |
| Peer-reviewed article | Okechi, (2025) | Nigeria | Community leaders’ perspectives on linking formal and informal health providers in Nigerian urban slums: a qualitative study |
| Peer-reviewed article | Onuh *et al.*, (2024) | Nigeria | Informal–Formal Healthcare Services Delivery Nexus in Nigeria’s Urban Slums: A Reconnaissance Study |
| Peer-reviewed article | Onwujekwe *et al.*, (2025) | Nigeria | Willingness to Pay for Improved Quality of Services from Informal Health Providers in Urban Slums in Nigeria |
| Peer-reviewed article | Onwujekwe *et al.*, (2011) | Nigeria | Do poor people use poor-quality providers? Evidence from the treatment of presumptive malaria in Nigeria |
| Peer-reviewed article | Ozor *et al.*, (2025) | Nigeria | Inequities in Household Out-Of-Pocket Spending Among Urban Slum Dwellers in Southeast Nigeria. |
| Peer-reviewed article | Scott-Emuakpor, (2013) | Nigeria | The evolution of health care systems in Nigeria: Which way forward in the twenty-first century |
| Peer-reviewed article | Sizear *et al.*, (2019) | Bangladesh | Perceptions of appropriate treatment among the informal allopathic providers: insights from a qualitative study in two peri-urban areas in Bangladesh. |
| Peer-reviewed article | Subedi, (2023) | Nepal | Legitimising traditional healers |
| Peer-reviewed article | Subedi, (2023b) | Nepal | Perspective Chapter: Integrating Traditional Healers into the National Health Care System – A Review and Reflection |
| Peer-reviewed article | Sudhinaraset *et al.*, (2013) | Multi-country | What Is the Role of Informal Healthcare Providers in Developing Countries? A Systematic Review. |
| Peer-reviewed article | Tenkorang (2016) | Ghana | Health provider characteristics and choice of health care facility among ghanaian health seekers. |
| Peer-reviewed article | Tiwari *et al.*, (2022) | Nepal | Health care seeking behavior for common childhood illnesses in Birendranagar municipality, Surkhet, Nepal: 2018 |
| Policy document | WHO (2015) | Bangladesh | Bangladesh health system review. |
| Media report/document | Yirenkyi, (2019) | Ghana | The Role of Traditional Medicine in Ghana Towards Achieving Universal Health Coverage: The Journey So Far |
